# A transdisciplinary process-oriented approach to evaluate infant exposure to indoor dust

**DOI:** 10.1038/s41370-025-00823-w

**Published:** 2026-01-14

**Authors:** Brandon E. Boor, Karen E. Adolph, Laura J. Claxton, Alexander Laskin, Orit Herzberg, Paige A. Thompson, Satya S. Patra, Brian H. Magnuson, Emily R. Halpern

**Affiliations:** 1 Lyles School of Civil and Construction Engineering, Purdue University, West Lafayette, IN USA; 2 Department of Psychology, New York University, New York, NY USA; 3 Department of Health and Kinesiology, Purdue University, West Lafayette, IN USA; 4 James Tarpo Jr. and Margaret Tarpo Department of Chemistry, Purdue University, West Lafayette, IN USA

**Keywords:** Early Life Exposure, Indoor Dust/House Dust/Dust, Metals, Exposure Modeling, Exposure Assessment, Emerging Contaminants

## Abstract

**Background:**

Infants are uniquely vulnerable to indoor dust exposure due to behaviors such as mouthing (hand- or object-to-mouth), crawling with hands on the floor, and close proximity to floor surfaces where dust accumulates. Dust acts as a reservoir for toxicants, including heavy metals, flame retardants, per- and polyfluoroalkyl substances, plasticizers, microplastics, and antimicrobials, which pose significant risks via ingestion and inhalation. Dust also contains allergens and microorganisms that may adversely impact respiratory health but have also been linked to protective immune effects, including a reduced risk of asthma and allergy development in early childhood. However, current exposure assessments lack a mechanistic understanding of how behavioral, developmental, and environmental factors interact to shape infant-specific dust exposure.

**Objective:**

To develop a transdisciplinary, process-oriented framework that integrates behavioral analysis, advanced dust characterization, and mechanistic modeling to evaluate infant exposure to indoor dust through ingestion and inhalation pathways.

**Methods:**

Videos of natural infant activity were collected from urban (New York, NY) and rural/suburban (West Lafayette, IN) homes to quantify mouthing frequency, duration, and other exposure-relevant behaviors across developmental stages. Dust samples were collected using standardized protocols and analyzed for surface concentrations, size distributions, morphological features, and toxicant profiles, focusing on heavy metals. Empirical datasets informed a mechanistic mass balance model to estimate size-resolved dust ingestion and inhalation rates.

**Results:**

Preliminary findings revealed variability in dust surface concentrations between carpets and smooth flooring, and differences in elemental concentrations between urban and rural/suburban homes. Dust size distributions (volume basis) revealed that nearly half of particles were smaller than 50 μm, with approximately 20% smaller than 20 μm, underscoring the role of finer particles in exposure pathways.

**Impact:**

- This study introduces a transdisciplinary, process-oriented framework to evaluate infant exposure to indoor dust, combining video-based behavioral analysis, advanced dust characterization, and mechanistic mass balance modeling. By linking developmental behaviors, environmental conditions, and dust properties, it will reveal key pathways driving ingestion and inhalation of dust-bound contaminants during infancy. These findings will advance exposure science by enabling more refined risk assessments and informing targeted strategies to protect vulnerable populations during critical stages of development.

## Introduction

Infants are uniquely vulnerable to exposure to environmental toxicants in indoor dust due to their behaviors, proximity to floor surfaces, and physiology [1–6]. Dust is a reservoir for toxicants, including heavy metals, and chemicals such as flame retardants, per- and polyfluoroalkyl substances, plasticizers, microplastics, antimicrobials, and other persistent organic pollutants [7–11]. Such toxicants accumulate on indoor surfaces from sources including cooking, combustion, building materials, and consumer products, and also infiltrate into homes from outdoor sources, such as vehicle and industrial emissions [12–14]. Dust also contains allergens and microorganisms of both outdoor and indoor origin, which may influence respiratory health in both harmful and protective ways [15–19].

Three primary pathways contribute to infant exposure to indoor dust: ingestion, inhalation, and dermal contact. Hand- and object-to-mouth behaviors are particularly prominent during infancy [20–23]. Video-based analyses reveal mouthing frequencies as high as 60 contacts per hour for young infants. Mouthing decreases with infant age but remains significant throughout early childhood [24–27]. Mouthing promotes dust ingestion from hands, toys, and other surfaces. Inhalation exposure occurs through resuspension of settled dust into the near-floor breathing zone, particularly during crawling [1–3, 28–32]. Robotic platforms and mass balance models show that crawling-induced particle resuspension creates a dense “personal cloud” of fine and coarse particles surrounding the infant [33]. The elevated concentrations of resuspended particles pose a significant inhalation risk, especially given the close proximity of the infant’s breathing zone to the floor. Dermal uptake of dust-bound toxicants also contributes to the total exposure burden, although it is less well characterized in early childhood [34–37]. This paper focuses on inhalation and ingestion pathways, which are comparatively better understood and more readily quantifiable through the behavioral and environmental measurements presented herein.

The physical and chemical characteristics of indoor dust influence exposure dynamics. Wide variations in the size, composition, and surface adhesion properties of dust particles affect ingestion and inhalation rates [1, 3, 12, 38–44]. Particle size distributions in residential dust commonly exhibit polydisperse profiles, with mass median diameters ranging from 50 to 100 μm, depending on collection and analysis methods [1, 10, 32, 45]. Surface dust concentrations differ based on home location (urban vs. rural/suburban) and flooring type, with higher levels typically found on carpets and area rugs compared to smooth, hard surfaces [18, 28, 31, 32, 46]. Variations in dust particle size and concentration underscore the need for size-resolved exposure assessments, as dust contact transfer fractions (or transfer efficiencies), surface adhesion forces, and resuspension dynamics are all size-dependent. The complex physicochemical properties of dust influence its capacity to bind pollutants, such as metals and organic chemicals, highlighting the need for detailed methods to characterize these interactions [7, 8, 47, 48]. Mass balance frameworks, such as that developed by Layton and Beamer [49], offer a valuable means of capturing the dynamic redistribution of indoor dust through deposition, resuspension, and track-in. These modeling approaches help elucidate how physical processes govern the spatial and temporal variability of dust-bound toxicants, supporting improved exposure assessments across diverse indoor environments.

Dust-bound contaminants have important implications for infant health. Heavy metals such as lead, cadmium, and arsenic, commonly detected in house dust, are associated with neurotoxicity, cognitive impairments, and anemia [11, 47, 50]. Polycyclic aromatic hydrocarbons, phthalates, and flame retardants contribute to oxidative stress, endocrine disruption, and respiratory dysfunction [7, 51–54]. Additionally, dust-bound microbial contaminants, including bacteria, fungi, and allergens, can exacerbate respiratory infections, allergic sensitization, and inflammatory responses [17, 18]. However, early-life exposure to diverse microbial content in household dust may also have protective effects against asthma and allergies, highlighting the complexity of early childhood dust exposure outcomes [19, 55]. The dual nature of early exposure highlights the importance of characterizing both the chemical toxicants and the biological components of dust that influence early childhood health outcomes.

Infant exposure to indoor dust is influenced by an interaction among environmental, physiological, developmental, and behavioral factors. Environmental factors, such as dust mass loading, particle size distributions, surface materials, and relative humidity, determine dust adhesion, contact transfer efficiency, and resuspension potential [1, 28, 30, 32, 56, 57]. For instance, carpets and area rugs tend to accumulate higher mass loadings of dust and generally exhibit greater resuspension fractions compared to smooth flooring, leading to elevated exposures in carpeted homes [28, 31, 58]. Physiological factors amplify exposure risks: infants’ higher respiratory rates, lower body mass, and close proximity of their breathing zone to the floor make them more susceptible to inhalation of resuspended dust particles relative to adults [1–3].

Developmental changes in infant behaviors play a key role in determining the nature and magnitude of indoor dust exposure. As motor skills develop, interactions with indoor surfaces shift. In particular, crawling leads to greater surface contact and the mobilization of settled dust into infants’ breathing zone compared with rolling, sitting, and walking. Similarly, crawling may contribute to dust ingestion due to increased hand-to-floor contact and frequent hand-to-mouth behavior. Crawlers also may be more likely to retrieve objects from the floor and bring them to their mouths [6, 59]. Thus, developmental changes in behavior are critical for evaluating infant-specific risks.

Despite decades of research on childhood exposures, significant knowledge gaps remain regarding the mechanistic processes governing infant dust ingestion and inhalation. Existing studies often rely on aggregate exposure estimates without accounting for size-specific dust properties, temporal variations in behavior, or real-world indoor microenvironments. Traditional methods, such as parent-report questionnaires and observational studies, fail to capture the complexity of dust transfer, resuspension, and behavior-driven exposure dynamics. Furthermore, the lack of integrated methods that bridge behavioral science, environmental engineering, and exposure modeling limit understanding of infant-specific exposure pathways. Addressing these limitations is critical to develop a mechanistic understanding of dust exposure based on behavioral data, dust characterization, and environmental modeling to mitigate risk during critical developmental windows.

To address these critical gaps, we introduce a transdisciplinary, process-oriented approach to evaluate infant exposure to indoor dust. Our approach integrates video-based behavioral analysis from developmental science, physical and toxicant characterization of dust from environmental science and analytical chemistry, and mechanistic mass balance modeling from environmental engineering to provide a comprehensive assessment of infant dust ingestion and inhalation pathways. The primary goal of this paper is to present an overview of this framework and its component methodologies. We report selected preliminary results solely to demonstrate the application and functionality of the framework. The methods and early findings presented here are part of an ongoing study funded by the U.S. Environmental Protection Agency. We detail procedures for quantifying infant behaviors, analyzing dust physical and toxicant properties, and modeling size-resolved dust ingestion and inhalation rates. Illustrative results reveal variability in dust surface concentrations and toxicant loads across diverse residential environments. While this study highlights these early findings, the presented framework is designed to be a generalizable approach for assessing size-resolved ingestion and inhalation exposure to indoor dust in early childhood. Additional manuscripts are in preparation to report more explicit results from the behavioral, compositional, and modeling components of this work.

## Overview of a transdisciplinary process-oriented approach to evaluate infant exposure to dust

**Fig. 1:**
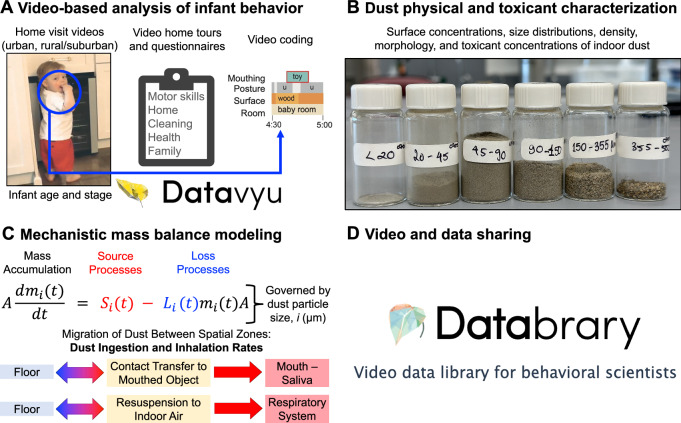
Transdisciplinary process-oriented approach to evaluate infant exposure to indoor dust via ingestion and inhalation pathways. **A** Video-based analysis of infant behavior during natural activities in the home. **B** Physical and toxicant characterization of indoor dust to evaluate variations in surface concentrations, size distributions, morphological features, and concentrations of heavy metals. **C** Mass balance modeling framework to mechanistically assess dust redistribution in infant near-floor microenvironments via contact transfer and resuspension, leading to intake through ingestion and inhalation pathways. **D** Open sharing of video data and results via the Databrary platform to enhance transparency, collaboration, and reproducibility in behavioral and exposure science research. Infant’s parent gave permission to use video clips and images of baby for research or educational purposes to online audiences in presentations and publications.

Our transdisciplinary approach provides a mechanistic evaluation of infant-specific exposure pathways, focusing on dust ingestion and inhalation. Our study includes three interconnected components that evaluate key factors driving indoor dust ingestion and inhalation in infants (Fig. 1):

### Video-based analysis of infant mouthing behavior during natural activity in the home

Infant behaviors are central to understanding how exposure occurs. Using video recordings collected during natural activity in the home, we quantify key exposure metrics such as mouthing frequency and duration. The analysis accounts for variations across developmental stages (e.g., rolling, sitting, crawling, and walking) and environmental conditions, such as floor type. High-resolution behavioral annotations use validated protocols to capture fine-scale interactions with surfaces and objects. These data provide a foundation for linking behaviors to dust contact transfer and resuspension dynamics.

### Dust physical and toxicant characterization

Indoor dust samples are collected during home visits using standardized vacuum-based protocols from key microenvironments. These samples are analyzed to determine surface mass loadings of dust, particle size distributions, and toxicant profiles, focusing on heavy metals (e.g., lead, arsenic). Using advanced analytical techniques (e.g., laser diffraction particle sizing, high-resolution mass spectrometry), we characterize dust properties that influence adhesion, transfer, and toxicity. This size-resolved and composition-specific analysis allows for the identification of dust fractions most relevant to ingestion and inhalation exposures.

### Mass balance modeling of infant exposure to indoor dust

Empirical data from behavioral analysis and dust characterization inform a mechanistic mass balance model to estimate infant exposure rates [1, 30]. The model incorporates size-resolved dust concentrations, object-specific contact transfer fractions, and resuspension fractions. Controlled laboratory experiments, including robotic simulations of crawling, provide additional data on dust contact transfer and resuspension dynamics. By integrating behavioral data and environmental measurements, the model estimates size-specific ingestion and inhalation rates across different developmental stages and microenvironments. This mechanistic approach accounts for temporal variability in behaviors and environmental factors, providing a robust foundation for exposure and risk assessments.

## Video-based analysis of infant mouthing behavior during natural activity in the home

We characterize infant mouthing behaviors and risk of dust ingestion during natural activity in the home.

### Open sharing of video data and annotations in the Databrary repository

To promote scientific transparency and reproducibility and to maximize public investments in research through data reuse, we requested caregiver permission to openly share data with authorized researchers in the Databrary digital video repository (databrary.org/support/irb). Caregivers could decline permission to share data with researchers outside the protocol, give permission only to authorized researchers on Databrary, or also give permission for short video excerpts to be shared publicly for educational purposes (databrary.org/support/irb/release-levels). At the end of the study, permissioned videos of infant-caregiver natural activity, behavioral annotation spreadsheets, video home tours, and demographic data will be shared with authorized Databrary researchers at databrary.org/volume/1364. Of the 176 sessions collected thus far, most caregivers in New York, New York (NY) (98%) and West Lafayette, Indiana (IN) (100%) formally agreed to share their data with authorized researchers in Databrary, and most (75% and 24%) also gave permission for excerpts to be shared. The video annotation manual, Datavyu scripts, de-identified processed data, analysis scripts, and, with caregiver permission, select video excerpts that illustrate procedures and findings will be publicly shared in the same volume.

### Data collection sites

**Fig. 2:**
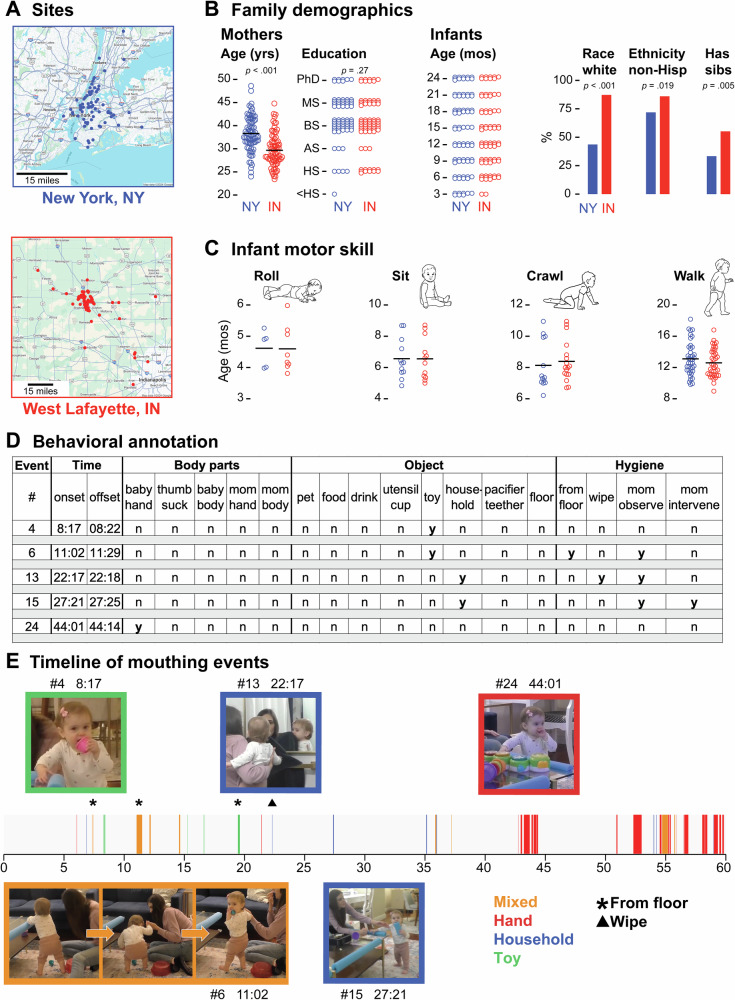
Video analysis of infant behaviors. **A** Data collection in urban New York, NY and rural/suburban West Lafayette, IN sites. Each pin represents one home. Most homes in NY were located in Manhattan and Brooklyn; most homes in IN were in the West Lafayette area. Data collection in IN covered a larger area than in NY because researchers in IN could drive to the homes (see map scale); however, travel time was approximately equal between sites. **B** Family demographics. Left panel shows mothers’ age and education for each site. Right panel shows infants’ age at the visit, race and ethnicity reported by the caregiver, and whether the infant had siblings living at home for each site. **C** Infants’ age at onset of most advanced motor skill at the visit for each site. Infants contributed data only to their most advanced skill. **D** Exemplar annotations from 5 of 60 mouthing events from one 12-month-old crawler. The Datavyu spreadsheet time-locks each user-defined event to its location in the video and data can be exported for analysis as shown in the figure. Onset and offset times are converted from frame numbers into minutes and seconds for purposes of illustration. Each row is a mouthing event and each column denotes information about whether particular body parts or objects were in or on the infant’s mouth. Hygiene information includes whether the body part and/or object had touched the floor in the 10 s prior to the mouthing event, whether the event was instigated by the caregiver to wipe the infant’s mouth/face, and whether the caregiver observed and/or intervened to prevent or interrupt the mouthing event. **E** Exemplar timeline of mouthing events from one 12-month-old crawler over the 60-min video from the home visit. The width of each bar denotes the duration of the event and bar color denotes what was mouthed; asterisks and triangles denote touching the floor and wiping, respectively. Photos show video frames from 5 illustrative events: #4 shows infant mouthing toy cup; #6 shows infant mouthing a toy that had recently come off the floor; #13 shows mom wiping infant’s face with a towel (household object); #15 shows infant mouthing a household object and the mom intervening; and #24 shows infant with her hand in her mouth. Infant’s parent gave permission to use video clips and images of baby for research or educational purposes to online audiences in presentations and publications.

We are collecting data at two sites—urban homes in the greater New York, NY area and rural/suburban homes in West Lafayette, IN and surrounding areas (see pins in Fig. 2A). Based on our preliminary sample that passed quality assurance (157/176 families), 69 homes (88.4%) in NY were apartments and multi-family housing in Manhattan, Brooklyn, and Queens; few homes had direct access to outdoor space (balcony, terrace, patio, lawn). Homes in IN were primarily single-family housing (95%)—75% in the neighborhoods surrounding Purdue University, 15% in rural areas, and 10% on farms; all had direct access to outdoor space.

### Family demographics

Families are recruited as convenience samples through social media, community events, and birth wards at local hospitals and receive $100 for participation. Inclusion criteria are infants’ age, term birth, birth weight of at least 2.3 kg, and no known disabilities; caregivers (mother or father) were at least 20 years of age when the infant was born and able to speak English to answer questionnaires (but parents did not have to speak English to their child).

Based on the data collected to date from sessions that passed quality assurance, caregivers had a wide range in age and education (Fig. 2B, left), mothers were older in the NY sample (*M* = 36.0, *SD* = 4.6) compared to the IN sample (*M* = 31.6, *SD* = 4.2), *t*(153) = 6.2, *p* < 0.001, but education did not differ across sites, χ^2^(5) = 6.4, *p* = 0.27. Infants were 3–24 months of age (sampled in 3-month intervals, within 2 weeks of their target age, target *n* = 12 per age at each site). At IN, infants’ race was primarily white, χ^2^(4) = 43.1, *p* < 0.001, and ethnicity at both sites was primarily non-Hispanic, χ^2^(1) = 5.5, *p* = .019. Most infants in NY were only children but half of the infants in IN had siblings, χ^2^(1) = 8.0, *p* = 0.005 (Fig. 2B, right).

### Infant motor skill

Parents reported infants’ motor skills: rolling (back to front), sitting (10 s unsupported), crawling (on hands and knees for 3 min independently without stopping or falling), and walking (3 min independently without stopping or falling). Figure 2C shows the age at onset for infants’ most advanced developmental stage at the time of the visit—infants who could roll but could not yet sit, crawl, or walk; infants who could sit but could not yet crawl or walk; infants who could crawl but could not yet walk; and infants who could walk. Age at achievement of motor skills did not differ between sites, all *p*s > 0.10.

### Procedure for home visits and quality assurance for adherence to protocol

Each home visit is scheduled at the caregivers’ convenience between infant meals and nap times. Caregivers are told that “we’re interested in learning how infants’ changing motor skills affect infants’ opportunities for learning and also how new motor skills might expose infants to potential safety hazards. A researcher will visit you and your baby in your home. You and your baby will be video recorded for one hour as you go about your day, followed by questions about your infant’s motor skills, health, and home environment. We will video record each room in your home to capture environmental opportunities for play and also potential safety hazards such as windows and stairs.” Although we do not explicitly mention infant dust ingestion, we say that, “We will collect dust samples from a few areas in your home.” Our impression is that caregivers do not clean in preparation for the home visit, and parents report the last time someone vacuumed the target dust collection areas.

During the home visit, a researcher records infants’ spontaneous behavior for one hour, during their natural, everyday activities, with a handheld digital video camera (30 fps), following procedures established by our research team (play-project.org) [60, 61]. Caregivers play with infants, do chores, work on their computer, talk on their phone, and so on. The researcher remains at the periphery of the room and does not interact with the infant or caregiver during filming.

An independent researcher conducts quality assurance on each one-hour video by ensuring that: the child meets inclusion criteria, the video recording represents natural activity in the home, and the video can be annotated. To pass quality assurance, the recording must be at least 50 min long after removing all instances when the infant was off camera; the caregiver took a bathroom break, breastfed the infant, or changed the infant’s diaper; the infant fell asleep or went outside; infant or caregiver interacted with researcher; or session was interrupted by a third person. In addition, the quality assurance manager checks the quality of the video (audio, lighting, camera angle) to ensure that the video can be annotated. Based on our sample to date, most (89.7%) of the one-hour natural activity recordings adhered to the protocol and passed quality assurance. Home visits (data not shown in Fig. 2) failed quality assurance due to not meeting inclusion criteria (*n* = 3), infant distracted by the researcher (*n* = 8), caregiver interacting with the researcher (*n* = 1), interactions with a third person (*n* = 5), and/or less than 50 min of annotatable data (*n* = 2).

### Video annotation of infant mouthing behavior and quality assurance on annotations

Researchers annotate infant mouthing behavior in the one-hour video of natural activity using Datavyu software (datavyu.org) that time locks user-defined annotations to their location in the video with frame-accurate precision (Fig. 2D). A primary ‘bulk’ coder identifies each instance when an object or body part was on or in the infant’s mouth and notes the first frame when the mouthing event began and the first frame when it ended (no mouthing for at least 1 s). Then—blind to the first coder’s annotations—a secondary ‘spot-checker’ annotates 25% of each one-hour video (in 5-min chunks distributed quasi-randomly across each 15 min of video). Every disagreement is automatically tagged via Datavyu scripts. Both coders meet after every few videos for quality assurance (e.g., to ensure inter-observer reliability). Typos and careless errors are fixed to avoid propagating known errors into final analyses, but true disagreements (e.g., one coder saw a mouthing event and the other did not) rely on the annotations of the primary coder. Based on 144 video files (randomly selected from the data in hand), the two coders agreed on the presence of mouthing in 97.9% of frames, Cohen’s κ = 0.94, *p* < 0.001, and agreed on the number of mouthing events, *r* = 0.91, *p* < 0.001.

In a second ‘pass’ on the quality-assured mouthing events, a Datavyu script inserts new cells with the onset and offset of each event. Then, as shown in the top panel of Fig. 2E, for each event, a primary coder determines whether the infant mouthed a body part (baby’s hand/fingers, thumb, or other body part; caregiver’s hand or other body part) or object (toy, household object, pacifier/teether, food, drink, eating utensil/cup, pet); the annotation system captured events when infants mouthed multiple things simultaneously or in quick succession within a single event (labeled ‘mixed’ in Fig. 2E). The coder also identifies several hygiene-related variables—whether the body part or object had touched the floor in the 10 s prior to the mouthing event, whether the event involved wiping the infant’s mouth, nose, or face (with a towel/rag, wipe, caregiver’s hand, etc.), and whether the caregiver observed or intervened (with words and/or actions) to prevent or interrupt the mouthing event. A secondary coder annotates 25% of each video to ensure inter-observer reliability. Based on 33 video files, the two coders agreed on 91.5% of what was mouthed, 97.2% of whom initiated the event, and 96.5% of events when the mouthed thing touched the floor, Cohen’s κ = 0.89–0.93, all *p*s < 0.001.

The bottom panel of Fig. 2E shows an exemplar timeline and video-frame captures from the video of one 12-month-old crawling infant over the one-hour session. Each vertical bar represents the duration of a single mouthing event, colors denote what was mouthed, asterisks denote whether the mouthed thing touched the floor in the prior 10 s, and triangles denote whether the event involved wiping the baby’s face.

## Indoor dust physical and toxicant characterization

This section details the collection, characterization, and analysis of indoor dust to evaluate its physical properties and contaminant profiles.

### Indoor dust collection protocol

**Fig. 3:**
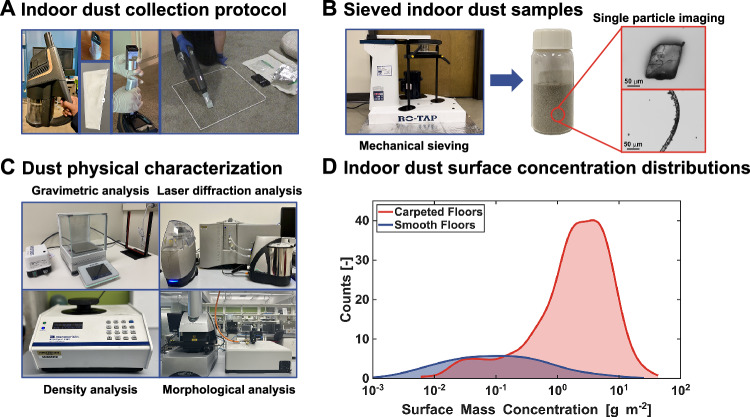
Indoor dust physical characterization. **A** Indoor dust collection protocol during home visits. **B** Mechanical sieving of indoor dust samples and single-particle imaging. **C** Analysis of dust mass concentrations via gravimetric methods, size distributions via laser diffraction analysis, density via gas pycnometry, and morphological features (e.g., sphericity, aspect ratio) via automated high-resolution microscopy. **D** Surface dust concentrations in sieved (≤2800 µm) indoor dust for carpeted and smooth flooring in urban homes (New York, NY) and rural/suburban homes (West Lafayette, IN).

Dust samples are collected using a standardized vacuum-based protocol (Fig. 3A) from three key microenvironments—living/common room, infant’s bedroom, and primary entryway—to quantify variations in dust surface concentrations, size distributions, and toxicant profiles. Entryways are prioritized for their role in tracking outdoor contaminants into the home [62].

A high-suction (100 kPa) handheld vacuum cleaner (Triflex Pro, Miele Inc.) equipped with a custom-built aluminum nozzle and triple-stitched nylon dust collection bags (10 µm pore size) ensures consistent and efficient dust recovery (Fig. 3A). The aluminum nozzle reduces particle loss due to electrostatic deposition and ensures effective sampling across smooth and carpeted surfaces. All vacuum components are cleaned with isopropyl alcohol wipes to prevent cross-contamination. Sampling procedures are tailored to flooring type. For carpeted floors, a 0.25 m^2^ area is divided into five sections, with dust collected using 30 systematic strokes. For smooth floors, a 0.5 m^2^ area is sampled with 60 strokes in a rectangular grid pattern to optimize recovery of lower dust loads. The vacuum cleaner is held vertically during shutdown to minimize dust fallout, and samples are sealed in pre-labeled Ziploc bags for transport. Field blanks are collected to monitor contamination. Video recordings of the dust collection process are collected for quality assurance. Protocol validation using indoor dust samples showed >95% total mass recovery efficiency in controlled laboratory tests.

### Video home tour

To contextualize dust accumulation and variability across homes, researchers conduct a detailed room-by-room video tour during each home visit. Videos capture all surfaces from ceiling to floor, including features such as fireplaces, bathroom/kitchen vents, heating, ventilation, and air conditioning (HVAC) systems, and windows. The videos also document all cleaning equipment (e.g., vacuum cleaners, brooms, and mops) and supplies (e.g., glass, surface, and floor cleaning products) to provide further insights into household maintenance practices.

### Indoor dust collection questionnaire

A caregiver report questionnaire is administered to capture environmental and behavioral factors influencing indoor dust accumulation and variability, including flooring materials (carpets, area rugs, hardwood, etc.). Cleaning practices are assessed, including vacuuming frequency, vacuum cleaner specifications (e.g., HEPA filters, bagged vs. bagless systems), sweeping, and mopping habits. Effective cleaning reduces dust surface concentrations, whereas improper methods, such as vacuums without HEPA filtration, can inadvertently redistribute dust particles into indoor air [1, 63].

Occupant behaviors, including indoor shoe-wearing and pet ownership, are also captured. Shoes introduce tracked-in outdoor contaminants such as heavy metals and pesticides, and pets contribute allergens, microbial content, and fur that increase indoor dust accumulation and biological complexity [15, 64]. Additional data on building characteristics, such as house age and potential legacy contaminants (e.g., lead and asbestos), are collected to identify risks associated with older homes [65]. Ventilation habits, including window-opening frequency and exhaust fan use, are documented alongside HVAC system type and maintenance practices. Finally, caregivers report qualitative indicators, such as visible dust, mold, and pet fur, to supplement the quantitative findings.

### Equipment and instrumentation for indoor dust physical and toxicant characterization

To obtain physical and toxicant characterizations of dust, we quantify surface loadings, size distributions, material densities, particle morphology, and toxicant profiles.

#### Mechanical sieving

The dust processing workflow (Fig. 3B) begins with mechanical sieving to achieve size-resolved classification of collected indoor dust samples. A stainless-steel sieve shaker (RO-TAP RX-29, W.S. Tyler) with a 2800 µm stainless-steel sieve isolates super-millimeter fractions (e.g., large fibers, food fragments, and debris) from finer dust fractions relevant to ingestion and inhalation. This initial fractionation ensures accurate separation, allowing particle size ranges important for exposure modeling to be analyzed in subsequent processes, such as laser diffraction particle sizing.

#### Gravimetric analysis

Surface dust mass concentrations are quantified with a high-precision analytical balance (±0.01 mg resolution) equipped with an antistatic kit to eliminate electrostatic interference (Fig. 3C). We create a comprehensive database of un-sieved and sieved dust surface mass loadings (expressed as g/m^2^ or mg/m^2^) for each home, providing critical baseline data to evaluate dust accumulation patterns across microenvironments. Following gravimetric analysis, sieved dust samples were subdivided for physical and chemical characterization. Sample homogeneity was assessed through visual inspection and periodic laser diffraction analysis. A subset of samples was used for supplemental analyses based on the collected dust mass at each location. Nominal sample masses were 80 mg for laser diffraction sizing, 10 mg for morphological analysis, and 80 mg for elemental analysis; pooling of dust across homes was not required.

#### Laser diffraction analysis

High-resolution dust particle size distributions are measured with a laser diffraction particle sizer (LDPS) (Mastersizer 3000, Malvern Panalytical Ltd.), capable of analyzing particles from 0.1 to 2500 µm (Fig. 3C). The instrument determines particle size through angular light scattering, applying Mie theory for fine particles and Fraunhofer diffraction for coarse particles. Dust samples sieved to 2800 µm are dispersed using distilled water with and without ultrasonication. Liquid dispersion protocols are validated using real indoor dust samples and standardized test dusts (e.g., ISO 12103-1 A1). Bulk refractive index corrections, obtained with an Abbe refractometer (Model C10, VEE GEE Scientific LLC), account for optical variations in indoor dust composition.

#### Density analysis

Determining dust particle material density is essential to convert size distributions from volume (via LDPS) to mass. Dust density is measured with a helium gas pycnometer (AccuPyc II 1340, Micromeritics Instruments Corp.), which calculates true density by measuring gas displacement in small-volume dust samples (Fig. 3C).

#### Morphological analysis

Dust particle morphology, including sphericity, aspect ratio, elongation, and circularity, is characterized with automated high-resolution optical microscopy (Malvern Morphologi G3SE-ID, Malvern Panalytical Ltd.) (Fig. 3C). These parameters influence dust adhesion, transport, and resuspension. The system’s single-particle imaging capability (Fig. 3B) provides detailed visualizations of particle shape and structure.

#### Elemental composition analysis

We focus on heavy metals of significant concern—lead (Pb), cadmium (Cd), arsenic (As), mercury (Hg), chromium (Cr), and vanadium (V)—which can accumulate in indoor dust through deteriorating paints, industrial emissions, household chemicals, contaminated soil, consumer products, and so on. Lead (Pb), for example, is a well-documented neurotoxicant that can cause cognitive impairment in infants, whereas cadmium (Cd) and arsenic (As) are associated with respiratory toxicity, immune suppression, and carcinogenic risks [8, 47, 65–67]. Mercury (Hg), often found in household products or legacy contamination, poses additional risks due to its bioaccumulative nature.

Dust samples are first analyzed using X-ray fluorescence (XRF) spectroscopy, a non-destructive technique that rapidly quantifies the relative atomic ratios of elements within the sample. XRF is particularly effective for screening metal elements and providing a broad compositional profile for heterogeneous dust matrices. For quantitative elemental analysis, sieved dust samples (≤2800 µm) undergo acid digestion and measurements using inductively coupled plasma mass spectrometry (ICP-MS), which delivers highly sensitive detection of trace elements, reporting concentrations down to the ppb range. The mass fraction of iron (Fe), quantified through ICP-MS, serves as a reference value and is combined with the relative elemental ratios from XRF to estimate mass fractions of elements not directly measured by ICP-MS. A limitation of our current approach is that elemental analysis was performed on bulk sieved dust (≤2800 µm) without further size fractionation and thus does not capture potential variation in toxicant concentrations across particle sizes, which remains an important direction for future work.

### Selected results: indoor dust physical and toxicant characteristics

#### Indoor dust surface concentrations and size distributions

Surface dust mass concentrations varied significantly across flooring types, with higher loadings observed on carpeted floors compared to smooth surfaces such as hardwood or tile (Fig. 3D). Among the 299 carpeted floor samples, surface concentrations ranged from 0.01 to 29.21 g/m^2^, with a mean of 3.48 g/m^2^ (median: 2.17 g/m^2^, interquartile range: 0.92 to 4.84 g/m^2^). In contrast, 79 smooth floor samples exhibited lower dust loads, ranging from 0.003 to 5.96 g/m^2^, with a mean of 0.46 g/m^2^ (median: 0.12 g/m^2^, interquartile range: 0.03–0.36 g/m^2^). These results confirm previous studies showing that carpets act as significant reservoirs for indoor dust accumulation due to their fibrous structure, which traps and retains particles, whereas smooth flooring surfaces promote lower dust retention due to easier cleaning [1, 2, 18, 28, 31, 32]. Carpets accumulate higher dust loads and retain particles for longer durations compared to smooth floors [18, 31, 58, 68]. These findings align with prior studies that highlight flooring material as a key factor influencing indoor dust migration dynamics and exposure pathways.

The particle size distributions of collected dust samples were analyzed using the LDPS [45, 69]. Results from selected urban and rural/suburban samples revealed that, by volume, approximately 6% of particles were smaller than 10 µm, 20% were smaller than 20 µm, and 50% were smaller than 50 µm, while 35% were larger than 100 µm. These findings align with prior studies showing that indoor dust consists of a polydisperse mixture of fine and coarse particles, reflecting contributions from outdoor soil, tracked-in debris, and indoor-generated particles [1, 18, 31, 70]. The presence of larger particles (>50 µm) underscores their role in hand-to-mouth ingestion pathways, particularly for infants frequently contacting floors and objects during crawling and mouthing activities. In contrast, the fine fraction (<10 µm) is relevant for inhalation exposure due to its ability to remain airborne and penetrate deep into the respiratory system.

#### Indoor Dust Elemental Composition and Geographical Variability

**Fig. 4:**
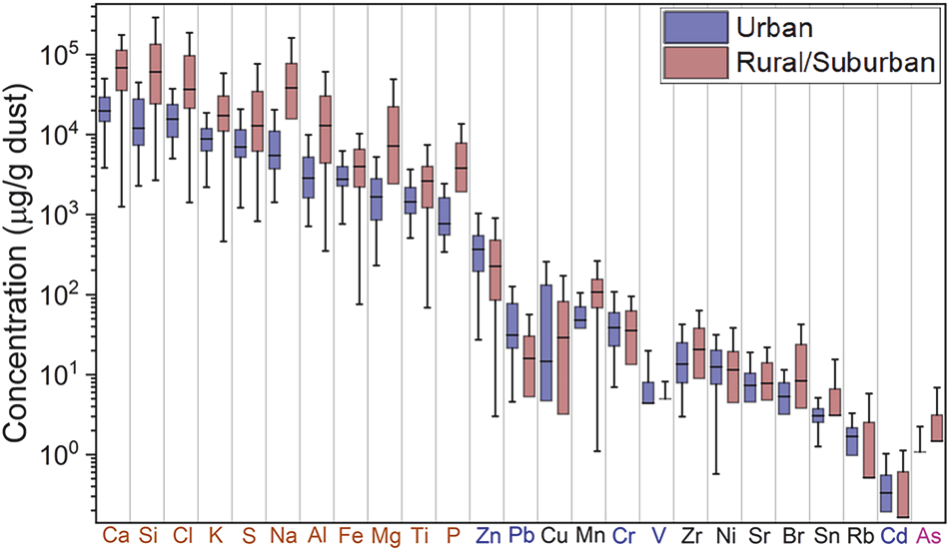
Indoor dust toxicant characterization. Box-and-whisker plots showing elemental mass fractions in indoor dust collected from urban homes in New York, NY, and rural/suburban homes in West Lafayette, IN. The vertical range of each box represents the interquartile range (25th to 75th percentiles), with the horizontal line indicating the median value. Whiskers extend to the 10th and 90th percentiles, respectively. The figure illustrates elemental mass fractions in indoor dust, highlighting differences between urban and rural/suburban samples. Earth-abundant elements, marked in brown, are more prevalent in rural/suburban samples and are primarily associated with outdoor soil and dust. Heavy metal toxicants, marked in blue, are linked to legacy sources typical of highly urbanized areas and are consequently more abundant in urban samples. Arsenic, marked in magenta, is another toxic element found at higher levels in rural/suburban samples, likely due to elevated geological arsenopyrite deposits reported in glacial and bedrock sediments near the West Lafayette, IN area.

Figure 4 presents all detected elements with mass fraction values exceeding 10^−1 ^µg/g, presenting average concentrations and statistical variability across urban dust samples from NY and rural/suburban dust samples from IN. Consistent with prior studies, the highest mass fractions were observed for earth-abundant elements (marked with brown color), measured at levels between 10^3^ and 10^5 ^µg/g, which are predominantly associated with outdoor soil minerals. These elements exhibited higher concentrations in IN, likely due to increased outdoor soil infiltration and more efficient indoor transport via occupant foot traffic. Metals of toxicological concern (Zn, Pb, Cr, V, Cd—marked by blue, and As marked by magenta) were detected at low mass fractions, consistent with levels reported in other household studies [66, 71–73]. Notably, Pb, V and Cd concentrations were approximately twice as high in urban samples, while As levels were three times higher in rural/suburban samples. The elevated Pb concentrations in urban dust are likely attributable to legacy sources common in highly urbanized areas, including deteriorating lead-based paint, historical deposits from leaded gasoline use, and the demolition or renovation of older materials containing lead (e.g., drywall, windows, and flooring) [65, 74, 75]. In contrast, the higher As levels observed in rural/suburban dust samples may be linked to elevated geological arsenopyrite deposits reported in glacial and bedrock sediments near the study area [76].

## Mass balance modeling of infant exposure to indoor dust

Accurately quantifying infant ingestion and inhalation of indoor dust requires a mechanistic understanding of how dust particles are transported and redistributed within the near-floor microenvironment where infants crawl, play, and explore. To address this, we developed a size-resolved mass balance framework to evaluate dust migration between spatial zones critical to exposure, including floors, indoor air, mouthed objects (e.g., hands, toys), saliva, and the respiratory system. This approach builds on the principle of mass conservation, where net dust accumulation within a spatial zone (e.g., flooring surfaces, indoor air) is determined by the balance between source processes delivering dust and loss processes removing it. For example, indoor air receives dust via resuspension from crawling and walking, while removal occurs through deposition, ventilation, and air filtration [1, 28, 31, 77]. By integrating behavioral observations, environmental measurements, and dust physical characterization, this framework enables size-resolved predictions of dust ingestion and inhalation exposures under real-world conditions.

The model addresses two primary components of dust exposure: ingestion and inhalation. For ingestion, it describes the transfer of size-resolved dust particles from flooring surfaces to mouthed objects, such as infants’ hands and toys, and subsequently to their mouths, where particles may be swallowed. Contact transfer fractions—quantifying the proportion of particles transferred per contact event—depend on dust particle size and morphology, surface properties (e.g., smooth versus carpeted flooring), contact pressure and duration, relative humidity, and films like saliva or skin oils [27, 56]. Controlled experiments, including robotic simulations and dust or soil adherence tests, provide size-specific contact transfer fractions, which integrate with behavioral metrics such as floor contact and mouthing frequencies from video analysis to estimate realistic dust ingestion rates using empirical and mechanistic approaches.

For inhalation exposure, the model accounts for dust resuspension triggered by infant locomotion, which generates mechanical disturbances and near-floor airflow turbulence, detaching settled dust particles. Similar to contact transfer fractions, resuspension fractions—quantifying the proportion of particles released into the air per disturbance—depend on dust particle characteristics, surface properties, and environmental factors [1, 31, 46, 77]. Crawling can release particles smaller than 20 µm into the infant breathing zone, forming dense dust clouds that elevate exposure risks [1–3]. Particles <10 µm are concerning as they remain airborne longer and penetrate deep into the respiratory system, while coarser particles >20 µm resettle quickly but dominate ingestion pathways [1, 78]. Chamber-based experiments provide size-specific resuspension data, which, combined with deposition rates, ventilation rates, and infant breathing parameters, enable robust predictions of inhalation exposure to resuspended dust. For example, Wu et al. [1] found that crawling-induced resuspension fractions varied from 10^−6^ to 10^−2^ across the 0.5–10 µm size range and among 12 different carpets, underscoring the strong influence of particle size and surface type on near-floor resuspension dynamics.

Infants experience simultaneous ingestion and inhalation of dust during locomotion and play. Traditional exposure models evaluate these pathways independently, overlooking their concurrent nature. As infants crawl, settled floor dust adheres to their hands and transfers to their mouth during mouthing events, while the same movement resuspends dust into the near-floor breathing zone, increasing inhalation exposure. Our unified mass balance framework links contact transfer and resuspension processes, enabling predictions of total dust exposure during dynamic activity. By integrating size-resolved particle processes with behavioral and environmental data, this framework comprehensively evaluates ingestion and inhalation pathways, capturing the complexities of indoor dust migration and redistribution in the home.

### Conceptual framework and mass balance equations

**Fig. 5:**
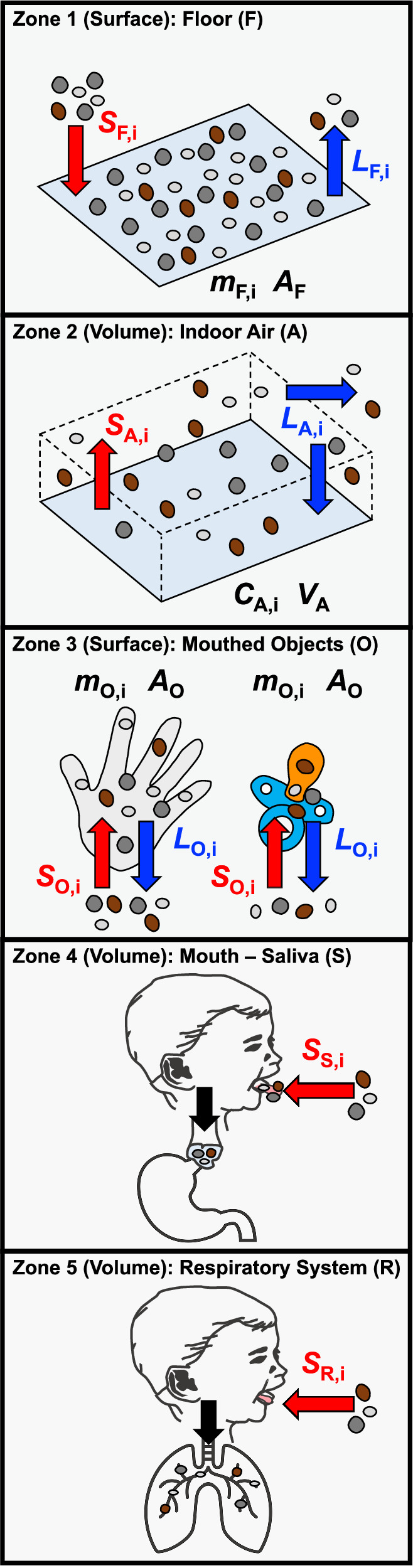
Schematic of mass balance modeling framework. Illustrated spatial zones include the floor, indoor air, mouthed objects, mouth–saliva, and respiratory system. Source (*S*) and loss (*L*) processes are represented as arrows delivering dust to a zone (source) or removing dust from a zone (loss). *i* denotes the particle size dependence of source and loss rates, while subscripts F, A, O, S, and R indicate each spatial zone. Dust mass concentrations (surface-based: *m* or volume-based: *C*) are defined for each zone’s area (*A*) or volume (*V*). Input parameters for source and loss terms are detailed in Table 1. Note: time dependency (*t*) of each term is not explicitly shown.

Figure 5 illustrates the five spatial zones modeled within this framework: Zone 1 (Surface): Floor (F), Zone 2 (Volume): Indoor Air (A), Zone 3 (Surface): Mouthed Objects (O), Zone 4 (Volume): Mouth–Saliva (S), and Zone 5 (Volume): Respiratory System (R). The framework applies the principle of mass conservation, represented through first-order differential equations, to account for the net accumulation of dust on surfaces (e.g., floors) and within volumes (e.g., indoor air). For surface zones, dust mass is calculated as the product of surface-based dust mass concentrations (*m*, g/m^2^ or mg/m^2^) and the surface area (*A*, m^2^). In volume zones, dust mass is expressed as the product of volume-based dust mass concentrations (*C*, mg/m^3^) and the corresponding volume (*V*, m^3^). Source terms (*S*, mg/h) represent processes that deliver dust to a given zone, such as resuspension from flooring surfaces into the indoor air zone. Loss terms (*L*, 1/h) represent removal processes, including deposition, ventilation, and air filtration, which remove dust particles from the indoor air zone [1, 28, 31, 49].

To capture the size-resolved nature of dust transport, accumulation, and removal, dust mass concentrations, source terms, and loss terms are treated as size-dependent variables, denoted with the subscript *i*. Coupled mass balance equations describe the dynamic movement of dust across spatial zones, with selected terms modeled as functions of time (*t*). For the floor, indoor air, and mouthed objects, these equations form the basis for predicting size-specific dust transport and exposure processes:

1 $$\begin{array}{cc}{{{\bf{Zone}}}}\,{{{\bf{1}}}}\,\left({{{\bf{Surface}}}}\right)\!\!:{{{\bf{Floor}}}}\,({{{\bf{F}}}})\!\!: & {A}_{F}\cdot \frac{d{m}_{F,i}\left(t\right)}{{dt}}={S}_{F,i}(t)-{L}_{F,i}(t)\cdot {m}_{F,i}\left(t\right)\cdot {A}_{F}\end{array}$$

2 $$\begin{array}{cc}{{{\bf{Zone}}}}\,{{{\bf{2}}}}\,\left({{{\bf{Volume}}}}\right)\!\!:{{{\bf{Indoor}}}}\;{{{\bf{Air}}}}\,({{{\bf{A}}}})\!\!: & {V}_{A}\cdot \frac{d{C}_{A,i}\left(t\right)}{{dt}}={S}_{A,i}(t)-{L}_{A,i}(t)\cdot {C}_{A,i}\left(t\right)\cdot {V}_{A}\end{array}$$

3 $$\begin{array}{cc}{{{\bf{Zone}}}}\,{{{\bf{3}}}}\,\left({{{\bf{Surface}}}}\right)\!\!:{{{\bf{Mouthed}}}}\; {{{\bf{Objects}}}}\,({{{\bf{O}}}})\!\!: & {A}_{O}\cdot \frac{d{m}_{O,i}\left(t\right)}{{dt}}={S}_{O,i}(t)-{L}_{O,i}(t)\cdot {m}_{O,i}\left(t\right)\cdot {A}_{O}\end{array}$$

The subscript (F, A, O) denotes the corresponding spatial zone. Dust mass concentrations are assumed to be uniform within each zone. For example, Zone 2, representing indoor air, is treated as well-mixed within the infant near-floor microenvironment to simplify mass balance calculations and ensure practical application for exposure assessment [1, 2, 30, 31]. While this assumption streamlines the model, each zone can be partitioned into sub-zones to capture spatial variations in dust concentrations, as demonstrated in recent work on infant crawling-induced dust resuspension [1]. The sizes of Zones 1 (floor surfaces) and 2 (indoor air) can be estimated using typical dimensions of living rooms and infant bedrooms. For simplified analyses, certain source and loss rate terms may be treated as approximately constant over time. *A*_F_ can be defined as the floor area available for resuspension or contact transfer.

Common size-resolved source and loss terms for Zones 1 to 3 are summarized in Table 1. For example, in Zone 1 (floor surfaces), sources include dust track-in, modeled as a net track-in rate (*T*_i_), and deposition of airborne particles, represented using a first-order deposition loss rate coefficient (*β*_i_). Loss terms account for dust removal via vacuuming or cleaning, modeled as a vacuuming/cleaning removal rate coefficient (*v*_i_); resuspension induced by infant locomotion, quantified using resuspension fractions (*r*_r,i_), defined as the fraction of dust particles on the floor surface that is in contact with the infant (e.g., foot, hand) and are released into the air by one stroke of a repetitive contact motion; and contact transfer to mouthed objects, described by contact transfer fractions (*ɸ*_FO,i_), defined as the fraction of dust particles on the floor surface that is in contact with an object and are transferred to the object by one contact of a repetitive contact motion. Inputs are drawn from chamber studies of dust redistribution (e.g., *β*_i_ and *r*_r,i_) [1, 28, 31], video analysis of infant behavior (e.g., *f*_OS_: object-to-mouth contact frequency), and dust physical characterization e.g., *m*_F,i_ from LDPS analysis of dust samples [24, 25]. Contact transfer and resuspension fractions vary by orders of magnitude with dust particle size, contact dynamics, and environmental conditions, underscoring the complexity of dust redistribution in infant near-floor microenvironments.

**Table 1:** Input parameters for mass balance modeling framework.

*S*_i_: Size-Resolved Source Processes (mg/h)	*L*_i_: Size-Resolved Loss Processes (1/h)
**Zone 1 (Surface): Floor (F)**	
**Track-in of dust:** ***S***_**F,i**_ = ***T***_**i**_, where: ***T***_**i**_ = net track-in rate (mg/h). **Deposition of airborne dust particles**: ***S***_**F,i**_ = ***β***_**i**_ **•** ***V***_**A**_ **•** ***C***_**A,i**_, where: ***β***_**i**_ = first-order deposition loss rate coefficient (1/h); ***V***_**A**_ = indoor air volume (m^3^); ***C***_**A,i**_ = indoor air volume-based dust mass concentration (mg/m^3^).	**Vacuuming/cleaning**: ***L***_**F,i**_ = ***v***_**i**_ **•** ***ε***_***v***_, where: ***v***_**i**_ = vacuuming/cleaning removal rate coefficient (1/h); ***ε***_***v***_ = fraction of floor area available for resuspension/contact transfer that is vacuumed/cleaned (-). **Infant locomotion-induced dust resuspension**: ***L***_**F,i**_ **=** ***r***_**r,i**_ **•** ***f***_**r**_ **•** ***ε***_**r**_, where: ***r***_**r,i**_ = resuspension fraction (-); ***f***_**r**_ = floor contact frequency (1/h); ***ε***_**r**_ = fraction of floor area available for resuspension/contact transfer that is in contact with infant (e.g., foot, hand) (-). **Dust contact transfer with mouthed object**: ***L***_**F,I**_ = ***ɸ***_**FO,i**_ **•** ***f***_**FO**_ **•** ***ε***_**FO**_, where: ***ɸ***_**FO,i**_ = floor-to-object contact transfer fraction (-); ***f***_**FO**_ = floor-to-object contact frequency (1/h); ***ε***_**FO**_ = fraction of floor area available for resuspension/contact transfer that is in contact with object (-).
**Zone 2 (Volume): Indoor Air (A)**	
**Infant locomotion-induced dust resuspension**: ***S***_**A,i**_ **=** ***r***_**r,i**_ **•** ***f***_**r**_ **•** ***ε***_**r**_ **•** ***m***_**F,i**_ **•** ***A***_**F**_, where: ***r***_**r,i**_ = resuspension fraction (-); ***f***_**r**_ = floor contact frequency (1/h); ***ε***_**r**_ = fraction of floor area available for resuspension/contact transfer that is in contact with infant (e.g., foot, hand) (-); ***m***_**F,i**_ = surface-based dust mass concentration on floor (mg/m^2^); ***A***_**F**_ = floor area available for resuspension/contact transfer (m^2^).	**Deposition of airborne dust particles**: ***L***_**A,i**_ = ***β***_**i**_, where: ***β***_**i**_ = first-order deposition loss rate coefficient (1/h). **Ventilation:** ***L***_**A,i**_ = ***λ***, where ***λ*** = ventilation rate (1/h). **Portable air filtration:** ***L***_**A,i**_ = ***η***_**i**_**/*V***_**A**_, where: ***η***_**i**_ = clean air delivery rate (m^3^/h); ***V***_**A**_ = indoor air volume (m^3^).
**Zone 3 (Surface): Mouthed Objects (O)**	
**Dust contact transfer with floor**: ***S***_**O,i**_ = ***ɸ***_**FO,i**_ **•** ***f***_**FO**_ **•** ***ε***_**FO**_ **•** ***m***_**F,i**_ **•** ***A***_**F**_, where: ***ɸ***_**FO,i**_ = floor-to-object contact transfer fraction (-); ***f***_**FO**_ = floor-to-object contact frequency (1/h); ***ε***_**FO**_ = fraction of floor area available for resuspension/contact transfer that is in contact with object (-); ***m***_**F,i**_ = surface-based dust mass concentration on floor (mg/m^2^); ***A***_**F**_ = floor area available for resuspension/contact transfer (m^2^). **Deposition of airborne dust particles**: ***S***_**O,i**_ = ***β***_**i**_ **•** ***V***_**A**_ **•** ***C***_**A,i**_, where: ***β***_**i**_ = first-order deposition loss rate coefficient (1/h); ***V***_**A**_ = indoor air volume (m^3^); ***C***_**A,i**_ = indoor air volume-based dust mass concentration (mg/m^3^).	**Washing/cleaning**: ***L***_**O,i**_ = ***ω***_**i**_ **•** ***ε***_**ω**_, where: ***ω***_**i**_ = washing/cleaning removal rate coefficient (1/h); ***ε***_**ω**_ = fraction of object area washed/cleaned (-). **Dust contact transfer with mouth (saliva)**: ***L***_**O,i**_ = ***ɸ***_**OS,i**_ **•** ***f***_**OS**_ **•** ***ε***_**OS**_, where: ***ɸ***_**OS,i**_ = object-to-mouth contact transfer fraction (-); ***f***_**OS**_ = object-to-mouth contact frequency (1/h); ***ε***_**OS**_ = fraction of object area in contact with mouth (-).
**Zone 4 (Volume): Mouth—Saliva (S)**	
**Dust contact transfer with mouthed object**: ***S***_**S,i**_ = ***ɸ***_**OS,i**_ **•** ***f***_**OS**_ **•** ***ε***_**OS**_ **•** ***m***_**O,i**_ **•** ***A***_**O**_, where: ***ɸ***_**OS,i**_ = object-to-mouth contact transfer fraction (-); ***f***_**OS**_ = object-to-mouth contact frequency (1/h); ***ε***_**OS**_ = fraction of object area in contact with mouth (-); ***m***_**O,i**_ = surface-based dust mass concentration on object (mg/m^2^); ***A***_**O**_ = object area (m^2^).	Not considered.
**Zone 5 (Volume): Respiratory System (R)**	
**Inhalation of resuspended airborne dust particles:** ***S***_**R,i**_ = ***C***_**A,i**_ **•** ***Q***_**R**_, where: ***C***_**A,i**_ = indoor air volume-based dust mass concentration (mg/m^3^); ***Q***_**R**_ = breathing rate (m^3^/h).	Not considered.

Summary of common size-dependent (*i*: dust particle size) source and loss processes relevant to indoor dust migration within infant near-floor microenvironments and associated dust ingestion and inhalation rates. Note: the time dependency (*t*) of each term is not explicitly shown.

For a typical infant locomotion or play event in the home, the model predicts dust mass concentrations on mouthed objects (*m*_O,i_) that contact the floor (e.g., hands and toys) and in indoor air (*C*_A,i_) following resuspension events. Dust ingestion rates and inhalation rates can then be estimated by incorporating incoming source terms for Zone 4 (saliva, *S*_S,i_, mg/h) and Zone 5 (respiratory system, *S*_R,i_, mg/h), as outlined in Table 1. For these zones, dust accumulation and loss terms are not considered. Ingestion rates are calculated by summing contributions from various objects, each characterized by size-resolved dust concentrations (*m*_O,i_), contact transfer fractions (*ɸ*_OS,i_, defined as the fraction of dust particles on the object surface that is in contact with the mouth and are transferred to the mouth by one contact of a repetitive contact motion), and contact frequencies (*f*_OS_). Inhalation rates incorporate size-specific airborne dust concentrations (*C*_A,i_) and age-specific breathing rates (*Q*_R_) [1, 2]. While size-integrated ingestion rates (*S*_S,i_ integrated over a specified particle size range) are well-documented [25, 79], data on size-resolved ingestion rates (*S*_S,i_) and inhalation rates for resuspended indoor dust (*S*_R,i_) remain limited. This mechanistic framework provides an integrated approach to quantify the likelihood of dust ingestion—via hand-to-mouth or object-to-mouth transfer—and dust inhalation through resuspension into the near-floor breathing zone. This integration underscores our transdisciplinary approach to comprehensively evaluating dust dynamics and exposure pathways within infant microenvironments.

### Controlled laboratory experiments to determine dust contact and resuspension fractions

**Fig. 6:**
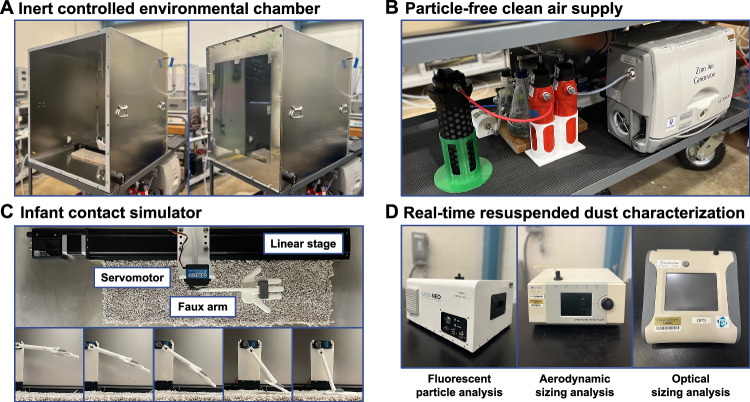
Laboratory experiments of dust contact transfer and resuspension. **A** Custom-built inert controlled environmental chamber with electropolished stainless steel surfaces. **B** Particle-free clean air supply delivered via a zero air generator and HEPA filtration. **C** Robotic infant contact simulator equipped with a linear stage for horizontal motion and a servomotor for rotational motion to replicate infant crawling dynamics. **D** Real-time analysis of resuspended dust mass concentrations using a wideband integrated bioaerosol sensor (WIBS), aerodynamic particle sizer (APS), and optical particle sizer (OPS), shown left to right.

To support development of size-resolved mass balance models for infant dust exposure, we are conducting controlled laboratory experiments to quantify dust contact transfer and resuspension fractions under infant-relevant conditions (Fig. 6). These experiments take place in a custom-built inert controlled environmental chamber (Fig. 6A) with a particle-free clean air supply to maintain near-zero background particle levels (Fig. 6B). A robotic infant contact simulator, mounted on a linear stage, replicates crawling motions with programmable contact time, frequency, impulse, and pressure to mimic infant locomotion (Fig. 6C). Flooring surfaces are seeded with indoor dust collected from homes. A suite of aerosol instruments, including an optical particle sizer (OPS), aerodynamic particle sizer (APS), and wideband integrated bioaerosol sensor (WIBS), continuously sample chamber air to measure real-time resuspended dust mass concentrations (*C*_A,i_), from which size-resolved resuspension fractions (*r*_r,i_) are estimated (Fig. 6D). In parallel, contact transfer fractions (*ɸ*_FO,i_) are estimated from floors to surrogate skin and toy surfaces. The robotic simulator performs controlled contact events with settled dust, and adhered particles are recovered and characterized using laser diffraction analysis. These measurements provide critical mechanistic inputs for estimating dust ingestion and inhalation rates within the mass balance framework.

### Toxicant dose via indoor dust ingestion

To illustrate potential health risks associated with indoor dust exposure, we calculated the average daily ingested dose (*ADD*, mg/kg/day) of heavy metals using the measured elemental concentrations from Fig. 4. While the size-resolved dust ingestion rates (*S*_S,i_) can be predicted through our mass balance framework, for this illustrative example, we used a common size-integrated (*S*_S_) literature value for the daily dust ingestion rate (200 mg/day [80]):

4 $${ADD}=\frac{E\,\cdot \,{S}_{S}}{{BW}}\times {10}^{-6}\,$$

where *E* is the measured mass fraction of heavy metals and other elements (µg/g), *BW* is the average infant body weight (here taken as 12 kg), and 10^−6^ is a unit conversion factor. For non-carcinogenic risk assessment, the hazard quotient (*HQ*) was calculated by dividing *ADD* values by element-specific reference dose (*RfD*) values [66]. Using the elemental concentrations from Fig. 4, the resulting *HQ* values were all below 1, indicating that acute adverse health effects are unlikely at these dust ingestion rates. Similarly, the lifetime cancer risk (*LCR*) was estimated as the product of *ADD* and element-specific cancer slope factors (*SF*). The calculated *LCR* values were below 10^−4^, which is within the range considered acceptable for carcinogenic risk.

This preliminary dose calculation demonstrates the potential utility of combining dust ingestion rates with measured elemental concentrations (Fig. 4) to estimate exposure risks. However, our project aims to advance beyond this simplified example by providing size-resolved (*ADD*_i_) and scenario-specific dose estimates. By integrating the mass balance framework with detailed environmental and behavioral data, we will enable more refined assessments that account for individual exposure scenarios and specific indoor environments, providing a comprehensive understanding of dust pollutant risks to infants.

## Conclusions and future directions

This study introduces a transdisciplinary, process-oriented framework to assess infant exposure to indoor dust, combining video-based behavioral analysis, advanced dust characterization, and mechanistic mass balance modeling. By capturing the intricate interplay between infant behaviors, dust properties, and environmental variables, this approach provides a foundation for understanding the key mechanisms driving dust ingestion and inhalation exposures. Future applications of this framework in diverse microenvironments—such as homes, daycare centers, and playgrounds—can yield deeper insights into developmental and behavioral factors influencing exposure, including floor contact frequency, object mouthing, and breathing zone proximity to dust-laden surfaces. Video data can be annotated using Datavyu software to systematically code these behaviors and shared via the Databrary platform, promoting transparency, accessibility, and collaboration across exposure science disciplines.

The methods outlined here, including advanced physical characterization of indoor dust using laser diffraction particle sizing and high-resolution microscopy, can be applied to evaluate dust particle size distributions and morphological features critical to dust transport, adhesion, and resuspension. Coupled with toxicant composition analysis through techniques such as high-resolution mass spectrometry, these data support the refinement of mass balance modeling frameworks to predict dust intake and toxicant dose via both ingestion and inhalation pathways. While dust inhalation pathways remain underexplored in traditional exposure studies, this framework emphasizes their inclusion alongside ingestion processes to provide a more comprehensive assessment. Key input parameters, including size-resolved contact transfer and resuspension fractions, can be derived from controlled laboratory experiments, addressing current data gaps and ensuring model accuracy for real-world exposure scenarios.

Moving forward, this transdisciplinary approach can be extended to additional contexts and populations to investigate how environmental conditions, dust particle size, and behavioral patterns interact to influence exposure dynamics. By fostering collaboration among behavioral scientists, environmental engineers, and chemists, future work will advance our understanding of infant-specific exposure pathways and their health implications. Continued integration of empirical data, advanced modeling, and cross-disciplinary methods will refine exposure estimates and inform public health interventions, particularly for vulnerable populations during critical developmental stages. As part of our ongoing efforts, this study highlights the importance of leveraging innovative methodologies to address complex exposure challenges and improve environmental health outcomes. Future work will expand the framework to evaluate dermal exposure pathways, enabling a more comprehensive assessment of total body burden in early childhood.

## Data Availability

Raw videos, behavioral annotations, and parent-report questionnaires are openly shared with authorized investigators on the Databrary platform (databrary.org/volume/1364).
